# Author response to “post-infection cognitive impairments in a cohort of elderly patients with COVID-19”

**DOI:** 10.1186/s13024-022-00565-5

**Published:** 2022-09-26

**Authors:** Yu-Hui Liu, Ye-Ran Wang, Qing-Hua Wang, Juan Liu, Yan-Jiang Wang

**Affiliations:** 1 Department of Neurology and Centre for Clinical Neuroscience, Daping Hospital, Third Military Medical University, Chongqing, China; 2 Chongqing Key Laboratory of Ageing and Brain Diseases, Chongqing, China; 3 Center for Excellence in Brain Science and Intelligence Technology, Chinese Academy of Sciences, Shanghai, China

To the Editor,

We appreciate the comments on our study about the 6-month cognitive outcomes of COVID-19 among older adults by Dr. Rahmouni Nesrine et al. In this study we found that COVID-19 survivors, especially those who survived severe infection, had worse cognitive performances than their uninfected spouses [1]. We agree with Rahmouni and colleagues that the methods used in our study have limitations. For the reason of feasibility during the pandemic, we chose uninfected spouses as the control. As their age, living conditions, and lifestyles were similar to those of patients, this control selection could help to reduce the bias attributed to these factors which are known to closely relate to cognitive functions. However, the choice of uninfected spouses as the control group would also cause some bias, because, as expected, uninfected spouses would certainly be in better health conditions. As pointed out by Rahmouni and colleagues, ICU admission was also found to be associated higher risk of dementia [2, 3], it is possible that ICU admission, but not severe COVID-19 itself, contributed to the long-term cognitive decline in severe cases. Subjects with pneumonia infected by non-COVID viruses and non-COVID ICU patients would be more appropriate as controls to examine the specific impact of COVID-19 on cognition.

As the aim of our study was to investigate whether COVID-19 could cause long-term cognitive decline, we did not use the cognitive assessment upon discharge as the baseline cognitive status, as it was post-infection status and the cognitive impairment was found to be common at acute stage of COVID-19 [4]. The pre-infection cognitive performance would be the ideal baseline cognitive status; however, this was not practical for our cohort. Therefore, the changes of cognition after infection were assessed with the Chinese version of the short form Informant Questionnaire on Cognitive Decline in the Elderly (IQCODE), which is often used to assess the longitudinal cognitive decline under circumstances where the baseline cognitive information is lacking [5]. The reporters of IQCODE questionnaire were informants who co-lived with the patients and their spouses, most of them were adult children of the patients who were familiar with the cognitive status of their parents. We did not collect the information about whether the informants were also infected with COVID-19, which is related to the reliability of their report on the cognitive change of their parents. We agree that sleep disorders after COVID-19 might generate impacts on the cognition of the survivors. This is an important issue and needs to be addressed to better understand the long-term impact of COVID-19 on cognition.

The COVID-19 pandemic has been profoundly plaguing our life and society and is still raging worldwide. The long-term health consequences of COVID-19 are an important public health issue but remain largely unknown at present time. We conducted this pilot study among the first bulk of patients in Wuhan COVID-19 pandemic and the data may not be entirely applied to the current COVID-19 patients as the virus has been mutated to Omicron strains with less toxicity. However, due to restrictions caused by the pandemic conditions, the methodology of our study has some limitations which might cause bias in interpreting the research findings. Emerging studies identified the long-term impacts of COVID-19 on both the structure and function of the brain [6–10]. In the future, the improvement of the methodology, more rigorous study design, and more intensive mechanistic investigations are needed to unveil the long-term impacts of COVID-19 on cognition and formulate the corresponding interventions to meet the challenge of the pandemic.

## Data Availability

Not applicable.
